# Chronic Noise Exposure Acts Cumulatively to Exacerbate Alzheimer’s Disease-Like Amyloid-β Pathology and Neuroinflammation in the Rat Hippocampus

**DOI:** 10.1038/srep12943

**Published:** 2015-08-07

**Authors:** Bo Cui, Kang Li, Zhihui Gai, Xiaojun She, Na Zhang, Chuanxiang Xu, Xuewei Chen, Gaihong An, Qiang Ma, Rui Wang

**Affiliations:** 1 Department of Occupational Hygiene, Tianjin Institute of Health and Environmental Medicine, Tianjin, China; 2 Shandong academy of occupational health and occupational medicine, Shandong academy of medical sciences, Jinan, China

## Abstract

A putative etiological association exists between noise exposure and Alzheimer’s disease (AD). Amyloid-β (Aβ) pathology is thought to be one of the primary initiating factors in AD. It has been further suggested that subsequent dysregulation of Aβ may play a mechanistic role in the AD-like pathophysiology associated with noise exposure. Here, we used ELISA, immunoblotting, cytokine arrays, and RT-PCR, to examine both hippocampal Aβ pathology and neuroinflammation in rats at different time points after noise exposure. We found that chronic noise exposure significantly accelerated the progressive overproduction of Aβ, which persisted for 7 to 14 days after the cessation of exposure. This effect was accompanied by up-regulated expression of amyloid precursor protein (APP) and its cleavage enzymes, β- and γ-secretases. Cytokine analysis revealed that chronic noise exposure increased levels of tumor necrosis factor-α and the receptor for advanced glycation end products, while decreasing the expression of activin A and platelet-derived growth factor- AA. Furthermore, we found persistent elevations of glial fibrillary acidic protein and ionized calcium-binding adapter molecule 1 expression that closely corresponded to the noise-induced increases in Aβ and neuroinflammation. These studies suggest that lifelong environmental noise exposure may have cumulative effects on the onset and development of AD.

People are increasingly exposed to environmental noise from many sources including work, traffic, media, and household appliances. Long-term noise exposure increases the risk of physical damage and is considered a health hazard^1^. Such exposure can have physiological or even pathological effects on the classical auditory system, as well as non-lemniscal brain regions such as the hippocampus and cerebral cortex. Studies therefore suggest that chronic noise exposure may induce abnormal auditory input to the brain resulting in aberrant changes in the hippocampus and cortex^2^,^3^,^4^,^5^,^6^,^7^. It has been reported, for instance, that such exposure results in the persistent tau pathology observed in Alzheimer’s disease (AD)^2^,^4^,^7^,^8^,^9^. In the hippocampus, excitotoxic and metabolic insults can ultimately result in memory loss^3^. Considering these findings, it is compelling to speculate that the environmental health hazard of noise exposure might be associated with an increased risk of developing AD.

Accumulation of β-amyloid peptide (Aβ), neuroinflammation, and prominent tau pathology in the hippocampus are important pathological features of AD. Aβ peptides are generated as a product of amyloid precursor protein (APP) sequential degradation by the APP-cleaving enzyme. An imbalance between the production, clearance, and aggregation of these peptides causes Aβ to accumulate, and is thought to be the initiating factor in AD. These processes can ultimately lead to neuronal metabolic failure and synaptic dysfunction^10^.

Recently, it has been shown in transgenic mouse models of AD that environmental stress can increase Aβ production and tau accumulation^11^,^12^. In addition, previous studies have shown in animal models that abnormal APP deposition occurs in neurons of the hippocampus, thalamus, and cerebral cortex following acute impulse noise exposure, and that the effects of such exposure were similar to those described in AD^13^,^14^. However, none of these studies determined whether chronic noise exposure has long-term after-effects on the accumulation of pathological Aβ peptides in the hippocampus, which might be critical to elucidating the etiological association between environmental noise and AD.

Numerous studies^2^,^7^,^15^ have found evidence of increased oxidative stress in the brain after noise exposure. Oxidative stress can accelerate the accumulation of amyloid and tau proteins in AD^16^,^17^. In addition, production of oxidizing free radicals, including reactive oxygen species and reactive nitrogen species, can be induced by increased cytokine production^18^,^19^,^20^,^21^. These findings led us to speculate that neuroinflammation may also play an important role in the noise-induced neuropathology associated with AD.

In the present study, we investigated the after-effects of chronic noise exposure on Aβ formation and neuroinflammation in the hippocampus, where neurodegeneration is the most prominent in AD. By answering these questions, we aimed to identify the potential associations between chronic noise exposure and the etiology underlying AD. Here we report that chronic noise exposure can cumulatively exacerbate AD-like Aβ pathology, which further results in neuroinflammation in the rat hippocampus.

## Methods

### Animal use and experimental grouping

A total of 96 8-week-old (200–220 g) male Wistar rats (obtained from the Lab Animal Center, Institute of Health and Environmental Medicine, Tianjin, P.R. China) were used in this study. The rats were kept in a room with a 12 h light/dark cycle (with lights on from 06:00 to 18:00) and controlled ambient temperature (23 ± 2 °C) and humidity (50–70%). The rats had free access to water and food in their cages and were allowed to habituate to the laboratory environment for 5 d prior to the start of the experiment. The rats were randomly assigned to either the noise-exposed or control group. Animals in the noise-exposed group were placed in an environment with 100 dB sound pressure level (SPL) white noise (4 h per d for 28 d, from 8:00 to 12:00). Rats in the control group were housed in similar cages but were exposed to background noise (below 40 dB SPL) from another chamber. At different time points (days 0, 3, 7, or 14) after the final exposure, both rats in the noise-exposed and control groups were sacrificed by decapitation for subsequent biochemical analyses (n = 12 rats per group and time point). All experiments were performed in accordance with approved guidelines specified by the Animal and Human Use in Research Committee of the Tianjin Institute of Health and Environmental Medicine.

### Noise exposure set-up

White noise was generated using a noise generator (BK 3560C, B&K Instruments, Denmark), amplified with a power amplifier (YONG-SHENG AUDIO P-150D, The Third Institute of China Electronic Technology Group, China), and delivered through a loudspeaker (ZM-16 S, Tianjin Zenmay Electroacoustic Equipment Co., Ltd., China). All exposures were performed as described in our previous study^4^.

### Determination of gene expression by real-time PCR

Hippocampal tissues from noise-exposed and control rats were removed at different time points following the last exposure and homogenized using a rapidly oscillating masher. Total RNA was extracted using an RNeasy Mini kit (TaKaRa Bio, Dalian, China) according to the manufacturer’s protocol. Total RNA was converted to cDNA by reverse transcription using a Transcriptor First Strand cDNA Synthesis Kit (TaKaRa Bio, Dalian, China). A primer pair designed to amplify the glyceraldehyde phosphate dehydrogenase (GAPDH) gene was used as an internal control. Specific primers and probes designed for rat APP, glial fibrillary acidic protein (GFAP), and GAPDH were used, as described in Table 1. Gene expression levels for APP and GFAP were assessed by quantitative real-time PCR (qPCR) performed under the following thermal cycling conditions: 2 min at 50 °C and 10 min at 95 °C, followed by 45 cycles at 95 °C for 5 s, and 57 °C for 30 s. Real-time PCR was performed using gene expression assays-on-demand and a Takara PCR Thermal Cycler Dice Real Time system (TaKaRa Bio, Dalian, China). The threshold cycle (Ct) of target genes was normalized to that of GAPDH. mRNA levels in noise-exposed animals were calculated after normalizing cycle thresholds to GAPDH expression, and are presented as fold-induction values (2^−ΔΔCt^) relative to those of control rats.

### Screening of inflammatory factors by protein array

Hippocampi from noise-exposed animals were compared with those from the control group at day 0, following the last exposure, using the 34 cytokine preconfigured RayBio^®^ Rat Cytokine Antibody Array G Series 2 (AAR-CYT-G2, RayBiotech, Norcross, GA, USA). The chip was read using a GenePix 4000B Microarray Scanner (Molecular Devices, Sunnyvale, CA, USA). Notably, the array was arranged in such a manner that each antibody was spotted twice, creating two replicates per protein of interest. Specific protocol details can be found at the RayBiotech, Inc. website (http://www.raybiotech. com). The layout of spotted primary antibodies is shown in Table 2.

### Determination of Protein Concentration by ELISA

Frozen rat hippocampi were homogenized in ice-cold 1 × phosphate buffered saline (0.02 mol/L, pH 7.0–7.2). Total protein concentrations were determined by the bicinchoninic acid method (Boster, Wuhan, China). Protein levels for Aβ1-40, Aβ1-42, β-secretase, γ-secretase, tumor necrosis factor α (TNF-α), receptor for advanced glycation end products (RAGE), platelet-derived growth factor AA (PDGF-AA), and activin A were determined in hippocampal homogenates using ELISA kits (BlueGene Biotech, Shanghai, China), according to the manufacturer’s recommendations (www.bluegene.cc). Concentration values of Aβ1-40, Aβ1-42, β-secretase, γ-secretase, TNF-α, RAGE, activin A, and PDGF-AA were normalized to total hippocampal protein levels. All concentrations were expressed in nanograms per gram of total protein, and were defined as the average of duplicates for a given rat.

### Western blot analysis

The western blot analysis was performed as previously described^22^. The primary antibodies used included the following: rabbit affinity-purified GFAP (polyclonal, 1:500; Boster Bio-Engineering Ltd. Co., China), rabbit affinity-purified ionized calcium-binding adapter molecule 1(IBA1) (polyclonal, 1:1000; Proteintech, USA), rabbit anti-β-actin (1:10000; Bioworld Technology, USA), and mouse affinity-purified β-amyloid (B-4, monoclonal, 1:500; Santa Cruz Biotechnology, USA), which recognizes APP and Aβ. The special secondary antibodies used were peroxidase-conjugated Affinipure goat anti-mouse and goat anti-rabbit IgG (ZSGB-BIO, Beijing, China).

### Statistics

All data were analyzed using SPSS 19.0 software (SPSS, Inc., USA). A one-way analysis of variance followed by Student’s *t*-test was used to determine statistical significance. Statistical significance levels were set to *p* < 0.05 for all tests. The data presented in the graphs indicate group means ± standard deviation.

## Results

### Chronic noise exposure leads to a persistent increase of Aβ in the hippocampus

**Figure 1:**
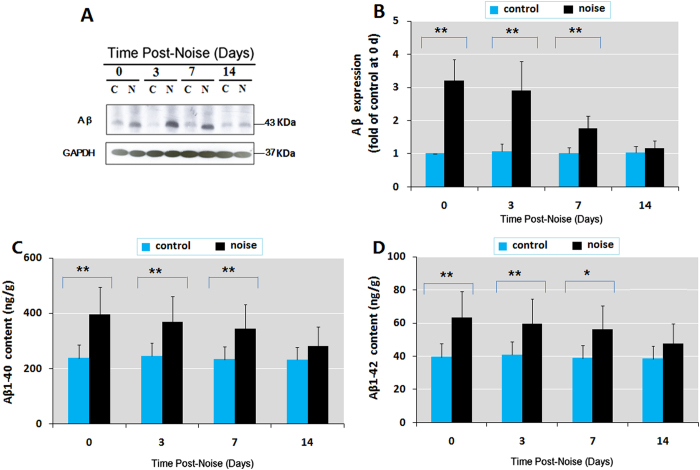
Chronic noise exposure leads to a persistent increase of Aβ in the hippocampus. (**A**) Western blot analysis of Aβ in the hippocampus under C (control) and N (chronic noise exposure) conditions. GAPDH was used as a loading control. (**B**) Quantification of immunoreactive band density measured in Panel A. Data are presented as the percent change relative to control samples. (**C,D**) Quantification of Aβ 1–40 and Aβ 1–42 levels by ELISA at different time points following noise exposure. Levels of Aβ 1–40 and Aβ 1–42 are shown as means ± standard deviation. *p < 0.05 and **p < 0.01, com*p*ared with respective controls by Student’s *t*-test (*n* = 6 per group).

In order to evaluate the effect of chronic noise exposure on the production of Aβ, we performed immunoblotting and ELISA assays to determine the relative levels of Aβ, Aβ1-40, and Aβ1-42 in hippocampus tissue. The expression of Aβ was significantly increased after 4 weeks of exposure to noise, with an increasing trend that persisted up to 7 days after the cessation of exposure (Fig. 1A,B). The amount of Aβ1-40 in the hippocampus was 1.7-fold higher than the amount in control rats at day 0 and decreased to 1.2-fold of this level at the end of observation period (Fig. 1C). Assessment of the Aβ1-42 content revealed a similar trend in the hippocampus after noise exposure (Fig. 1D).

### Chronic noise exposure facilitates the expression of APP, β- and γ- secretase

**Figure 2:**
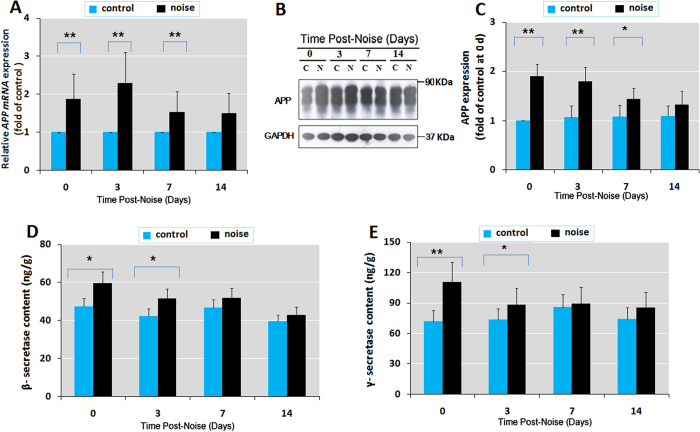
Chronic noise exposure facilitates the expression of APP, β-, and γ- secretase in the hippocampus. (**A**) Comparison of APP mRNA expression levels in control and noise-exposed rats by quantitative real-time PCR. (**B**) Western blot analysis of hippocampal APP expression under C (control) and N (chronic noise exposure) conditions. GAPDH was used as a loading control. (**C**) Quantification of immunoreactive band density measured in Panel B. Data are presented as the percent changes relative to control samples. (**D,E**) Levels of β- and γ- secretase were quantified at different time points after noise exposure by ELISA. Bars represent means ± S.D. *p < 0.05 and **p < 0.01, compared with respective controls by Student’s *t*-test (*n* = 6 per group).

Aβ is generated from the sequential cleavage of APP by β-secretase and γ-secretase, constituting the major source of this protein in the brain. In order to explore the effect of chronic noise exposure on the production of Aβ, we determined the protein and mRNA levels of APP by western blot and RT–PCR analyses, respectively. We also estimated β-secretase and γ-secretase protein levels by ELISA. RT–PCR analysis showed that there was a significant increase in the expression of APP mRNA in the hippocampus at days 0, 3, and 7 following noise exposure; this trend largely decreased by day 14 (Fig. 2A). Western blot analysis also showed a robust increase in APP protein levels in the noise-exposed group at days 0, 3, and 7 after exposure (Fig. 2B,C). In addition, the ELISA results showed that β-secretase protein significantly increased by an average of 26.7% and 21.7% at days 0 and 3, respectively (Fig. 2D). By days 7 and 14 following exposure, β-secretase levels declined to baseline (Fig. 2D). There was also an increase in γ-secretase protein by an average of 54.5% and 20.5% at days 0 and 3, respectively; these levels declined to baseline at days 7 and 14 after the end of exposure (Fig. 2E). These results suggest that chronic noise exposure leads to Aβ accumulation by facilitating the expression and subsequent β-/γ-cleavage of APP.

### Chronic noise exposure causes abnormal inflammatory changes in the hippocampus

**Figure 3:**
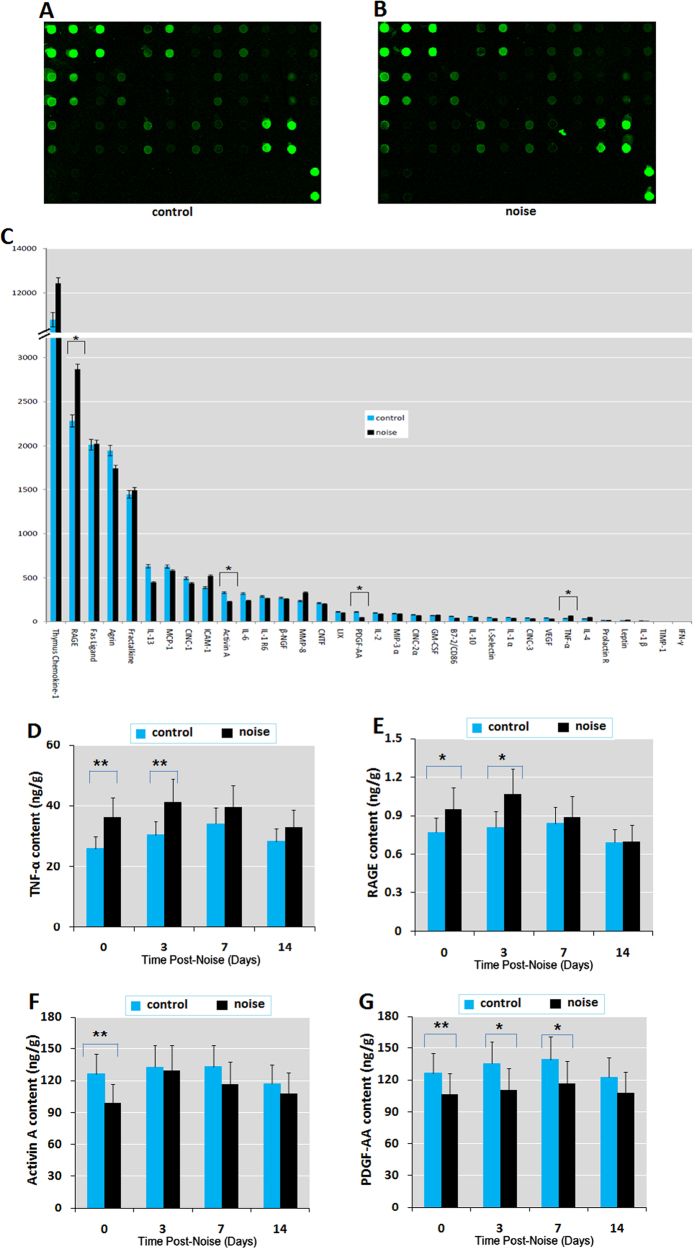
Chronic noise exposure causes abnormal inflammatory changes in the hippocampi of rats at different time points following noise exposure, as measured by protein array and ELISA. (**A,B**) Protein array format. Image representative of control **(A**) and noise-exposed (**B**) cytokine arrays in the Cy3 channel. A key to the location of spotted primary antibodies is shown in Table 2. (**C**) Normalized net intensity of 34 cytokines in hippocampi of control and noise-exposed rats at day 0 following noise exposure. Bars represent means ± S.D. *p < 0.05, compared with matched controls by Student’s *t*-test (*n* = 4 per group). (D-G) ELISA analysis showing the levels of cytokines following noise exposure. Bars represent means ± S.D. *p < 0.05 and **p < 0.01, compared with respective controls by Student’s *t*-test (*n* = 6 per group).

To better understand the neuroinflammation induced by chronic noise exposure, we examined the hippocampi of rats for alterations in cytokines using the inflammatory cytokine antibody array, followed by ELISA analysis. A schematic of the chip format and representative image of control and noise-exposed arrays in the Cy3 channel are shown in Fig. 3A,B. When we directly analyzed hippocampal contents by antibody array analysis, two of the 34 cytokines examined (TNF-α and RAGE) were expressed more abundantly in noise-exposed rats than controls, and two (activin A and PDGF-AA) were expressed less in noise-exposed rats than in the control group (Fig. 3C).

Our finding suggests that noise exposure may influence the local cytokine environment in the hippocampus. To further explore this effect, we compared expression profiles of key pro-inflammatory cytokines between noise-exposed and control rats in the hippocampus at different time points. Using an ELISA array kit, we found that noise exposure-induced increases in TNF-α and RAGE and corresponding reductions in activin A and PDGF-AA persisted for at least 3 to 7 days after cessation of noise exposure (Fig. 3D–G). These data suggest that chronic noise exposure may cause neuroinflammation in the hippocampus, one of the most important structures initially implicated in AD-like neuropathology.

### Chronic noise exposure facilitates the activation of neuroglia in the hippocampus

**Figure 4:**
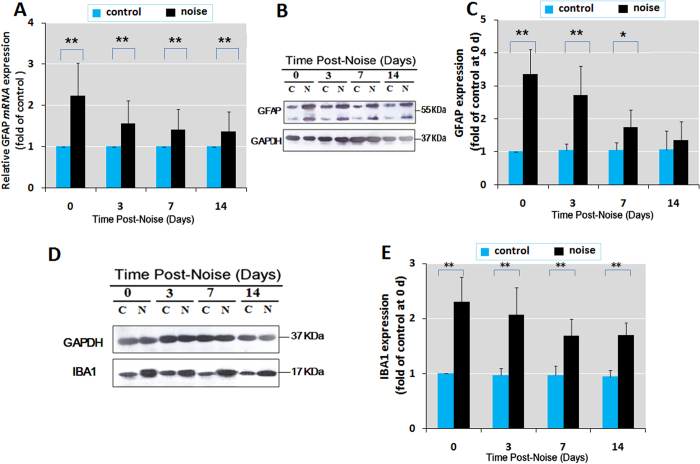
Chronic noise exposure causes persistent overexpression of GFAP in the hippocampus. (**A**) Comparison of GFAP mRNA expression levels at different time points in control and noise-exposed rats by quantitative real-time PCR. (**B,D**) Western blot analysis of GFAP and IBA1 under C (control) and N (chronic noise exposure) conditions at different time points. GAPDH was used as a loading control. The immunoreactive band density was quantified and presented as the percent change relative to control samples (**C,E**). Bars represent means ± S.D. *p < 0.05 and **p < 0.01, compared with respective controls by Student’s *t*-test (n = 6 per group).

Glial activation is one of the pathological hallmarks of neurodegenerative diseases including AD. IBA1 and GFAP are markers for microglia and astroglia, respectively, that can be used to detect neuroglial activation during neuroinflammation. Significant increases in GFAP mRNA expression were observed in the hippocampus at days 0, 3, 7, and 14 following noise exposure (Fig. 4A). Quantitative immunoblotting analysis also revealed that changes in GFAP protein levels were similar to those of GFAP mRNA over the course of the observation period, with the exception of GFAP expression at day 14, which decreased to the control level (Fig. 4B,C). Western blot analysis also showed a significant increase in IBA1 protein levels in the noise-exposed group at days 0, 3, 7 and 14 after exposure (Fig. 4D,E).These data suggest that chronic noise exposure may cause long-lasting neuroglial activation in the hippocampus, playing an important role in the initiation of neuroinflammation.

## Discussion

Recent work has implicated environmental influences in the increased risk for development of AD^23^,^24^,^25^. For instance, a relationship has been demonstrated between noise exposure and various indicators of AD-like neuropathology including tau hyperphosphorylation, cognitive deficit, excitotoxicity, and oxidative stress^2^,^3^,^4^,^7^,^8^,^9^,^15^. Results of another study further suggest that chronic noise exposure increases the risk of developing AD^26^. The present study delivers new insights into the link between noise exposure and AD. Our results represent a comprehensive description of the *in vivo* after-effects of noise exposure on both Aβ accumulation and neuroinflammation in rats. Our study also, for the first time, implicates neuroglia in noise-induced augmentation of AD-like pathology. In addition, this is the first study to report noise-induced changes in pathological amyloid accumulation and neuroinflammation in the rat hippocampus.

Aβ peptides are natural products of metabolism that consist of 36 to 43 amino acids. Aβ42 is toxic to cells and is much more prone to lead to aggregation and subsequent damage than monomers of Aβ40^27^,^28^. Aβ peptides originate from proteolysis of the APP by the sequential enzymatic actions of β-secretase and γ-secretase^29^. These peptides can grow into fibrils, which arrange into β-pleated sheets to form the insoluble fibers found in advanced amyloid plaques. The finding that chronic psychological stress induces Aβ accumulation has been well established in a large body of research^30^,^31^,^32^,^33^. Stress induced from acute impulse noise was found to increase the deposition of APP in neurons located in the cerebral cortex, thalamus, and hippocampus^13^,^14^. Our results demonstrate that a persistent increase in the levels of Aβ1-40 and 1-42 and the expression of APP and β-/γ-secretase occurs in the hippocampi of rats following chronic noise exposure. These findings suggest that long-term noise exposure may have a cumulative impact on the onset and progress of Aβ pathology, increasing the risk of neuropathological damage.

An imbalance between the production, clearance, and aggregation of peptides, causes Aβ to accumulate, which may be the initiating factor in AD. Cerebral plaques laden with Aβ and neurofibrillary tangles in medial temporal-lobe structures are important pathological features of AD. Recent evidence has shown that the presence of Aβ triggers glycogen synthase kinase 3 (GSK3β) activation, which causes tau hyperphosphorylation^34^. Our recent study found that chronic noise exposure caused long-term increases in the proportion of phosphorylated tau in both soluble and insoluble fractions^4^, with the latter showing delayed onset. Considering these findings, the hypothesis is that chronic noise exposure is implicated in the progression from physiological reactions to pathological changes in AD is compelling. It therefore seems highly plausible that noise-induced dysregulation of tau phosphorylation and accumulation of Aβ in the hippocampus could manifest as neuronal impairment, which subsequently contributes to the development of neurodegenerative diseases.

There is increasing evidence that neuroinflammation and stress-induced pathophysiology are related events^35^,^36^. Proinflammatory cytokines are known to be elevated in several neuropathological states associated with deficits in learning and memory, and can amplify the generation of additional cytokines, glutamate, and oxidative stress^19^,^21^. Furthermore, inflammation can increase Aβ production while decreasing its clearance^37^,^38^. In the current study, we observed a significant elevation in the levels of TNF-α, and RAGE in the hippocampus of noise-exposed rats, indicative of enhanced inflammation in the primary brain structure involved in learning and memory. Significant enhancement of these two important inflammatory factors in response to 4 weeks of noise-induced stress confirms previous findings that inflammation can be induced in the brain in response to various types of stressors^39^,^40^,^41^,^42^. Experimental studies have reported that TNF- α can cause inhibition of long-term potentiation (a form of neuronal plasticity widely believed to underlie learning and memory) in the dentate gyrus of the rat hippocampus^43^. Recently, it was demonstrated that the blockade of TNF synthesis decreased multiple hallmark features of AD, including Aβ accumulation and deficits in memory function, while preserving levels of synaptophysin^44^. Aβ is also able to trigger the activation of RAGE, facilitating the progression of neurodegeneration. RAGE can reciprocally bind Aβ, transduce extracellular Aβ toxic and inflammatory effects and mediate influx of vascular Aβ, thereby amplifying the generation of cytokines, glutamate, and nitric oxide^10^,^19^,^21^.

Our data also showed a significant decrease in the levels of two neuroprotective cytokines in the hippocampus of noise-exposed rats: activin A and PDGF-AA. Activin A is a homodimer of inhibin β_A_, which belongs to the transforming growth factor-β superfamily^45^. Recent studies reveal novel and important functions of activin in the processes of tissue repair, inflammatory disease, and modulation of synaptic plasticity in various brain regions including the hippocampus^46^,^47^. Recent advances have also shed light on neuroprotective actions of activin in the hippocampus during excitotoxic impairment: activin A has been found to act as an anti-inflammatory factor essential for neurogenesis in the adult CNS following excitotoxic neurodegeneration^48^,^49^. In addition, the application of recombinant activin shows neuroprotective effects from toxic insults in cultured hippocampal neurons^50^. PDGF dimers bind to two classes of PDGF receptors, which activation indirectly inhibits N-methyl-D-aspartate (NMDA) currents by modifying the cytoskeleton^51^. Furthermore, PDGF is also neuroprotective in hippocampal slices and cultured neurons^52^,^53^. Thus, we suggest that the noise-induced increase of proinflammatory factors (TNF- α and RAGE) and corresponding decrease of the anti-inflammatory factors (activin A and PDGF-AA) may cause a flux of Aβ across the blood-brain barrier^10^. This, in turn, may increase amyloid accumulation, attenuate neuroprotective effects, and exacerbate neuroinflammatory pathology. In combination, these events may eventually result in the development of AD.

Activated microglia and reactive astrocytes are a primary source of inflammation in AD. Up-regulation of these cells in inflammatory response has been observed in brain regions associated with high levels of AD pathological markers including the frontal cortex and limbic system^54^. During chronic inflammation, microglia generate reactive oxygen species (ROS) and release signals to recruit peripheral immune cells for an inflammatory response^55^. GFAP was up-regulated in the hippocampus of sound-stressed rats, which indicates excessive astrocyte activation^56^. Our present observation that noise exposure increased GFAP and IBA1 expression indicates that such exposure could aggravate neuroglial activation in the brain, in turn mediating a RAGE-dependent neuroinflammatory response^57^. Because pro-inflammatory cytokines and astrocytes reciprocally activate one another, this process could ultimately result in a positive feedback loop that can become self-sustaining and pathological in nature.

Hyperphosphorylated tau accumulation in the hippocampus and cortex and cognitive impairments are well known consequences of chronic noise exposure. Although the cellular mechanisms are still poorly understood, accompanying events to these processes include dysregulation of NMDA receptor signaling and increases in oxidative stress^2^,^3^,^4^,^7^,^8^,^9^,^15^. It has been reported that that Aβ causes tau hyperphosphorylation by activating p38 or GSK3β signaling^34^. Our previous work has demonstrated that chronic noise exposure triggers the formation of insoluble hyperphosphorylated tau in AD-related brain regions, concurrent with the dysregulation of GSK3β and phosphatases^4^. Thus, the results of the present study, together with these previously reported results provide a mechanistic sequence through which chronic noise exposure leads to the expression of AD-related pathological markers.

The work of Sotiropoulos *et al.* has shown that exposure to long-term stress acts cumulatively to precipitate AD-like pathology and cognitive deficits in transgenic animals^58^. Our demonstration that chronic noise exposure can induce AD-like pathology in non-transgenic rats reinforces the view that repeated noise exposure over a long period may be an etiological factor in the development of AD. In this respect, the growing body of evidence for the association between chronic noise exposure and AD^26^ is worth noting.

Our combined results suggest that chronic noise exposure may increase Aβ accumulation and induce a pro-inflammatory response that includes TNF-α and RAGE overexpression in the hippocampus. Our findings also implicate the involvement of neuroglia dysregulation in noise-induced AD-like pathology. These studies provide novel experimental evidence in support of the hypothesis that an etiological association exists between chronic noise exposure and the pathophysiology of AD. These findings further suggest that lifelong environmental exposure to noise may have a cumulative effect on the onset and development of AD.

## Additional Information

**How to cite this article**: Cui, B. *et al.* Chronic Noise Exposure Acts Cumulatively to Exacerbate Alzheimer’s Disease-Like Amyloid-β Pathology and Neuroinflammation in the Rat Hippocampus. *Sci. Rep.* **5**, 12943; doi: 10.1038/srep12943 (2015).

## Figures and Tables

**Table 1:** Rat primers used for real-time RT-PCR.

Gene	Primers
APP	F: 5′-AAGTGCGCCCCATTCTTTTA-3′
	R: 5′-AAAGTTGTTCCTGTTGCCGC-3′
GFAP	F: 5′-AGTGGTATCGGTCCAAGTTTGC-3′
	R: 5′-TGGCGGCGATAGTCATTAGC-3′
GAPDH	F:5′-GGCAAGTTCAATGGCACAGT-3′
	R:5′-TGGTGAAGACGCCAGTAGACTC-3′

APP, (β-amyloid precursor protein); GFAP, (glial fibrillary acidic protein); GAPDH, (glycolytic glyceraldehyde-3-phosphate dehydrogenase).

**Table 2:** Primary antibody map.

POS 1	POS 2	POS 3	NEG	Activin A	Agrin	B7-2/CD86	beta-NGF	CINC-1	CINC-2 alpha	CINC-3	CNTF
POS 1	POS 2	POS 3	NEG	Activin A	Agrin	B7-2/CD86	beta-NGF	CINC-1	CINC-2 alpha	CINC-3	CNTF
Fas Ligand	Fractalkine	GM-CSF	ICAM-1	IFN-gamma	IL-1 alpha	IL-1 beta	IL-1 R6	IL-2	IL-4	IL-6	IL-10
Fas Ligand	Fractalkine	GM-CSF	ICAM-1	IFN-gamma	IL-1 alpha	IL-1 beta	IL-1 R6	IL-2	IL-4	IL-6	IL-10
IL-13	Leptin	LIX	L-Selectin	MCP-1	MIP-3 alpha	MMP-8	PDGF-AA	Prolactin R	RAGE	Thymus Chemokine-1	TIMP-1
IL-13	Leptin	LIX	L-Selectin	MCP-1	MIP-3 alpha	MMP-8	PDGF-AA	Prolactin R	RAGE	Thymus Chemokine-1	TIMP-1
TNF-alpha	VEGF	NEG	NEG	NEG	NEG	NEG	NEG	NEG	NEG	NEG	POS 1
TNF-alpha	VEGF	NEG	NEG	NEG	NEG	NEG	NEG	NEG	NEG	NEG	POS 1

Antibody layout for each array on the microarray chip (RayBio^®^ Rat Cytokine Antibody Array G series 2; image adapted from RayBiotech^**®**^). Each primary antibody was spotted twice per array; eight arrays were spotted per chip. Positive and negative controls were used to determine signal variation between arrays.
